# Conservation physiology of plants

**DOI:** 10.1093/conphys/cou007

**Published:** 2014-02-25

**Authors:** Mark van Kleunen

**Affiliations:** Ecology, Department of Biology, University of Konstanz, Universitätsstrasse 10, D-78457 Konstanz, Germany

Conservation physiology was first identified as an emerging discipline in a landmark paper by Wikelski and Cooke (2006). They defined it as ‘the study of physiological responses of organisms to human alteration of the environment that might cause or contribute to population decline’. Although the case studies and examples presented by Wikelski and Cooke (2006) focused on wild animals, they also indicated that conservation physiology should be applicable to all taxa. With the launch of the journal with the same name, 1 year ago, this taxonomic inclusiveness was made more explicit, and the definition was broadened to ‘an integrative scientific discipline applying physiological concepts, tools and knowledge to characterizing biological diversity and its ecological implications; understanding and predicting how organisms, populations and ecosystems respond to environmental change and stressors; and solving conservation problems across the broad range of taxa (i.e. including microbes, plants and animals)’ (Cooke *et al.*, 2013).

Although the definition of conservation physiology, and also the journal, covers all taxa, plants (and also microbes and, among animals, the invertebrates) are still clearly under-represented. Of the 32 papers that were published in the journal in 2013, only three (9%) focused on plants (Funk, 2013; Hay and Probert, 2013; Lambers *et al.*, 2013). This under-representation of plants, however, appears to be a general trend in conservation science, because the journal *Conservation Biology* had only 10 of 93 contributed papers (11%) focusing on plants in 2013. The journal *Biological Conservation* did a bit better, with 59 of 309 regular papers (19%) focusing on plants in 2013. Given the importance of plants as primary producers, which are indispensable for all other organisms, and the fact that 10 065 of the 21 286 species (47%) assessed by the IUCN Red List as globally threatened are plants (http://cmsdocs.s3.amazonaws.com/summarystats/2013_2_RL_Stats_Table1.pdf, last assessed 28 December 2013), they clearly deserve more attention in the field of conservation physiology and conservation science in general.

Conservation science has many important, frequently intertwined, sub-disciplines, including conservation policy, conservation genetics and conservation physiology (Cooke *et al.*, 2013). The strength of physiology, and thus of conservation physiology, is that it focuses on the mechanisms underlying patterns by identifying cause-and-effect relationships, preferably through experimentation (Cooke *et al.*, 2013). Physiology is directly related to the functioning and function of plants. This means that physiological knowledge is imperative for understanding the habitat requirements of endangered native plants and of potentially invasive exotic plants, as well as the impacts of invasive exotic plants and migrating native plants. An accessory advantage of working with plants is that they lend themselves extremely well to experimental study, because they are sessile, can be marked easily and, frequently, can be grown in large numbers in greenhouse or garden conditions. Plants are thus ideal objects for conservation physiological studies.

Given that plants are under-represented, it is logical to ask, what kind of plant studies fit within the scope of conservation physiology? The three reviews on plants that were published in *Conservation Physiology* in 2013 do a great job in setting the scene. Lambers *et al.* (2013) reviewed the research on phosphorus-sensitive plants in a global biodiversity hotspot. Many of these species are threatened by the introduced pathogen *Phytophthora cinnamomi* and by eutrophication; the latter partly due to large-scale application of phosphite-containing fungicides (biostats) that are used to fight the pathogen. This illustrates how one conservation measure (use of fungicide to protect native plants from an introduced pathogen) may cause undesirable side-effects (eutrophication). Physiological understanding of how phosphite functions could help in the development of alternative fungicides with fewer negative side-effects. Hay and Probert (2013) reviewed recent research on seed conservation of wild plant species. They clearly make the case that if we want to preserve genetic material of wild plant species in *ex situ* seed banks for conservation purposes, physiological research is imperative for developing optimal storage, germination and growth conditions. Funk (2013) reviewed research on physiological characteristics of exotic plant species invading low-resource environments. Prevention of and mitigation of the impacts of such invasions require physiological research that resolves the question of whether exotic species manage to invade low-resource environments through enhanced resource acquisition, resource conservation or both. These three reviews thus already illustrate three important plant-related topics in conservation physiology, i.e. causes of threat to native plants, *ex situ* conservation and invasive exotic plants.

An important topic that has not yet been covered in *Conservation Physiology* is how plants will respond to climate change. Given that physiology underlies the fundamental niche of a species, physiological studies can inform predictive models on potential responses of plants to climate change. Related topics are how endangered and invasive plant species will respond to increased CO_2_ levels and how their vulnerability to diseases may change in novel climatic conditions. Furthermore, as we seem to fail miserably in reducing greenhouse-gas emissions, it also becomes more likely that governments will start to implement geo-engineering methods to reduce incoming solar radiation or atmospheric CO_2_ levels (Caldeira *et al.*, 2013). Undesired ecological side-effects of these methods will raise novel conservation issues, for which physiological knowledge will be imperative. Other topics that have not yet been covered are the physiological responses of plants to pollution, as well as how endangered species that are difficult to propagate from seeds could be multiplied using tissue culture or other techniques. Although the list of potential topics mentioned in this article is far from exhaustive, I hope it illustrates that many of the plant-related topics on which many of us work already or will work in the future fit within the scope of *Conservation Physiology*.
